# Alpha-1,2-Mannosidase and Hence N-Glycosylation Are Required for Regulatory T Cell Migration and Allograft Tolerance in Mice

**DOI:** 10.1371/journal.pone.0008894

**Published:** 2010-01-26

**Authors:** Elaine T. Long, Stephanie Baker, Vanessa Oliveira, Birgit Sawitzki, Kathryn J. Wood

**Affiliations:** Transplant Research Immunology Group, Nuffield Department of Surgery, University of Oxford, John Radcliffe Hospital, Oxford, United Kingdom; New York University, United States of America

## Abstract

**Background:**

Specific immunological unresponsiveness to alloantigens can be induced in vivo by treating mice with a donor alloantigen in combination with a non-depleting anti-CD4 antibody. This tolerance induction protocol enriches for alloantigen reactive regulatory T cells (Treg). We previously demonstrated that alpha-1,2-mannosidase, an enzyme involved in the synthesis and processing of N-linked glycoproteins, is highly expressed in tolerant mice, in both graft infiltrating leukocytes and peripheral blood lymphocytes.

**Principal Findings:**

In this study we have identified that alpha-1,2-mannosidase expression increases in CD25^+^CD4^+^ Treg when they encounter alloantigen *in vivo.* When alpha-1,2-mannosidase enzyme activity was blocked, Treg retained their capacity to suppress T cell proliferation *in vitro* but were unable to bind to physiologically relevant ligands *in vitro*. Further *in vivo* analysis demonstrated that blocking alpha-1,2-mannosidase in Treg resulted in the migration of significantly lower numbers to the peripheral lymph nodes in skin grafted mice following adoptive transfer, where they were less able to inhibit the proliferation of naïve T cells responding to donor alloantigen and hence unable prevent allograft rejection *in vivo*.

**Significance:**

Taken together, our results suggest that activation of alloantigen reactive Treg results in increased alpha-1,2-mannosidase expression and altered N-glycosylation of cell surface proteins. In our experimental system, altered N-glycosylation is not essential for intrinsic Treg suppressive capacity, but is essential *in vivo* as it facilitates Treg migration to sites where they can regulate immune priming. Migration of Treg is central to their role in regulating *in vivo* immune responses and may require specific changes in N-glycosylation upon antigen encounter.

## Introduction

Glycosylation involves the addition and removal of carbohydrate moieties to newly synthesized proteins orchestrated by a sequence of enzymes in the Golgi and endoplasmic reticulum [1]. It is a highly regulated process and specific oligosaccharides can alter both protein stability and function. Asparagine (N)-linked glycans are one kind of carbohydrate moiety found on cell surface glycoproteins; divided into high mannose-, hybrid- and complex-type according to the sugar component and the structure of sugar chains linking to the common oligosaccharide core (Man_3_GlcNAc_2_) [2]. There is considerable evidence that N-glycans play a key role in immune regulation [1].

N-glycosylation is tightly controlled during both the differentiation and activation of T lymphocytes and determines the ability of T cells to respond to extracellular stimuli and mediate cell-cell interactions [1], [3], [4], [5], [6], [7]. Ablation of the glycosyltransferase Mgat5 leads to increased TCR signaling and autoimmune disease *in vivo*, due to the loss of N-glycans that mediate the interaction of TCR molecules with galectins and hence restriction of TCR clustering [3]. Removal of N-glycosylation sites in the TCR constant domain can also increase the avidity of the TCR [8], which is being explored as a strategy to target cancer cells. Activation of mouse CD4^+^ and CD8^+^ T cells leads to dramatic remodeling of terminal glycosylation patterns of N-glycans which may alter the recognition of activated and resting T cells by other cell types expressing glycan-binding proteins that recognize terminal sequences of N-glycans [9].

Alpha-1,2-mannosidase is an enzyme involved in the synthesis and maturation of N-glycoproteins, where it successively removes mannose residues from Man_9_GlcNAc_2_ to generate Man_5_GlcNAc_2_ [10]. Our laboratory identified alpha-1,2-mannosidase as a marker that is highly expressed during induction and maintenance of operational tolerance to alloantigens *in vivo* resulting in allograft acceptance of both kidney and heart grafts, in two species, rat and mouse [11]. Alpha-1,2-mannosidase (Entrez GeneID: 17155) mRNA shows a strong positive correlation with graft function and decreases in both peripheral blood leukocytes and graft infiltrating leukocytes prior to rejection, suggesting that it may be useful marker for monitoring allograft function in clinical transplantation [11].

Achieving immunological tolerance to donor alloantigens without the need for long-term administration of immunosuppressive drugs is a major goal in transplantation. Regulatory T cells (Treg) comprise a subset of T lymphocytes that can suppress immune responses, control immune responsiveness to donor alloantigens, and have the potential to play a role in both inducing and maintaining transplant tolerance *in vivo* [12]. In animals expressing alpha-1,2-mannosidase, we have shown that immunological unresponsiveness to alloantigen is dependent on Treg [13] and that alpha-1,2-mannosidase mRNA is upregulated in Treg when they re-encounter alloantigen *in vivo*. Here we have investigated the hypothesis that alpha-1,2-mannosidase function and hence N-glycosylation, is required for Treg function and migration *in vivo*. We show that alpha-1,2-mannosidase function is not required for the suppressive capacity of Treg *in vitro*, but influences Treg adherence *in vitro* and migration *in vivo*. Defects in migration of Treg treated with kifunensine (KIF) that specifically inhibits the catalytic activity of alpha-1,2-mannosidase [14], [15], results in their impaired ability to prevent effector T cell priming and hence rejection of allogeneic skin grafts. These data suggest that upon alloantigen encounter, increased alpha-1,2-mannosidase and hence N-glycosylation are important for Treg function as they facilitate their transit *in vivo* to sites where they can suppress T cell activation leading to tissue pathology, as demonstrated in this model by rejection of donor allografts.

## Results

### Alpha-1,2-Mannosidase Expression Increases in Activated Alloantigen Reactive Treg

**Figure 1:**
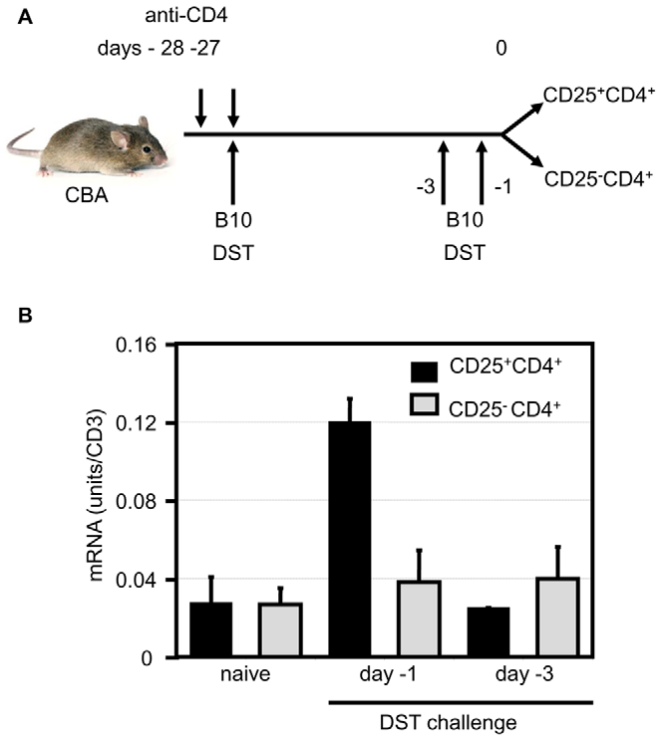
Alpha-1,2-mannosidase expression is increased in CD25^+^CD4^+^ cells after alloantigen re-challenge *in vivo.* a) CBA were pre-treated with an anti-CD4 mAb at days -28 and -27 (200 µg YTS 177). At day -27, mice also received an allogeneic blood transfusion (250 µl B10, DST). At days -3 or -1, mice received a DST reboost. CD25^+^CD4^+^ and CD25^−^CD4^+^ cells were purified from the spleen of these animals at day 0. RNA was extracted from these cells and cDNA was synthesized. (b) Alpha-1,2-mannosidase expression was assessed by Real Time RT-PCR. CD3 expression was measured to normalise the cDNA concentration in each sample. Error bars represent the standard deviation. The data presented are representative of 4 separate experiments.

T cell-mediated processes including activation and homing are accompanied by changes in cell surface N-glycosylation which result in an N-glycan signature [9]. Alpha-1,2-mannosidase is a key enzyme involved in directing this process of N-glycosylation. We have shown previously that alpha-1,2-mannosidase is upregulated in graft infiltrating leukocytes from long-term surviving heart grafts following pre-treatment of mice with donor alloantigen (DST) under the cover of anti-CD4 therapy (177) [11]. CD25^+^CD4^+^ Treg with the capacity to prevent skin allograft rejection are generated following this 177/DST protocol [13], [16], [17]. Therefore, we wanted to determine whether alloantigen-reactive Treg upregulate alpha-1,2-mannosidase upon antigen encounter. Following pre-treatment of mice with the 177/DST tolerance induction protocol, either one or three days before harvest mice received an alloantigen DST reboost to reactivate alloantigen reactive T cells *in vivo*, as shown in Figure 1a. Although only a small fraction of cells are believed to be donor-specific, we have previously detected significant differences in mRNA expression by Treg following re-stimulation with donor alloantigen using this protocol [18]. Treg or CD25^−^CD4^+^ cells were purified from the spleens of these mice by FACS sorting and alpha-1,2-mannosidase mRNA was quantified by real-time PCR, normalized to CD3. Figure 1b shows approximately a four-fold increase in alpha-1,2-mannosidase mRNA expression in CD25^+^CD4^+^ cells 24h after DST. This increase is transient as expression returns to basal levels 3 days after alloantigen exposure, and is not observed in CD25^−^CD4^+^ cells.

### Expression of N-Glycans Increases on the Cell Surface of Activated Treg

**Figure 2:**
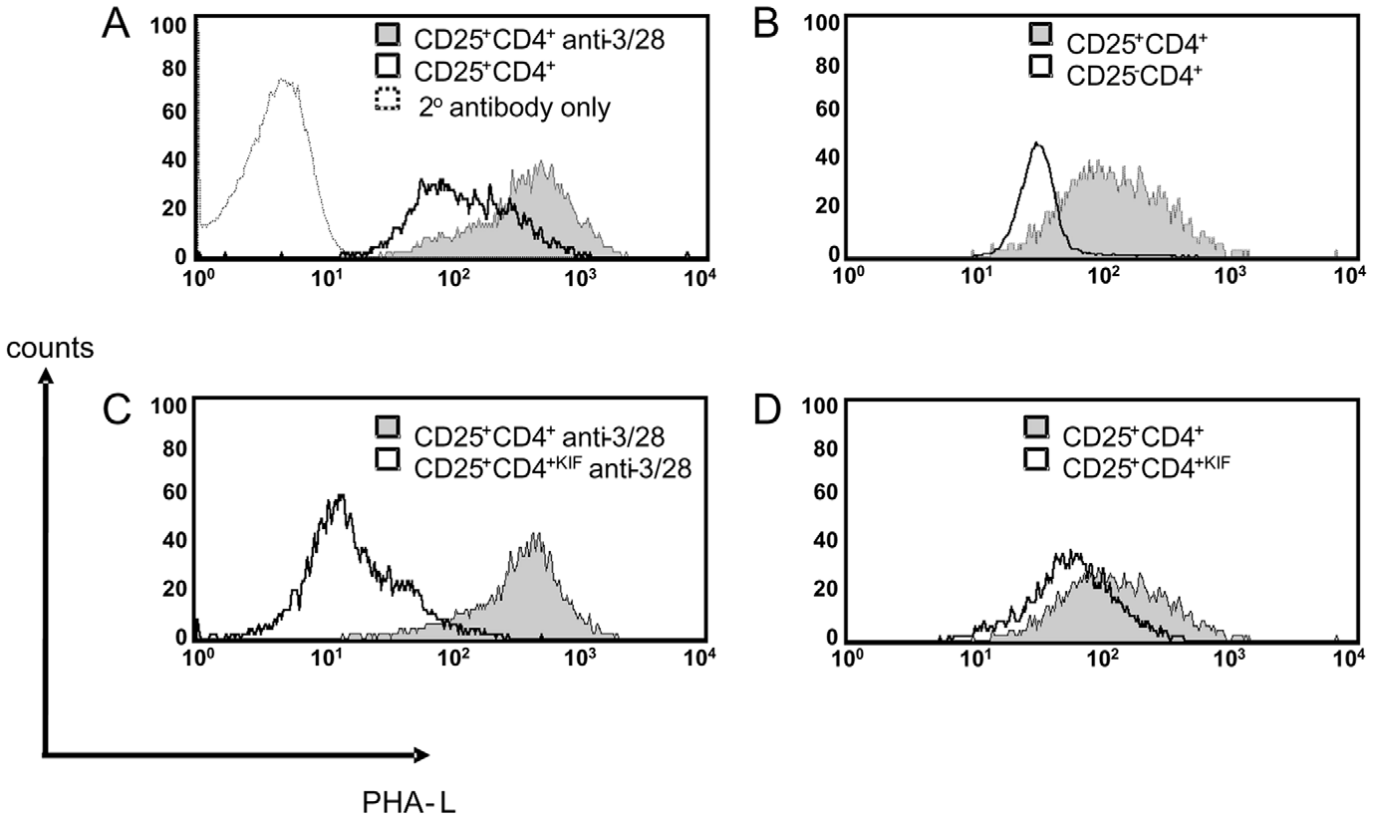
Surface N-glycosylation levels increase in activated CD25^+^CD4^+^ T cells. Total CBA splenocytes or purified cells in culture were stained with PHA-L and surface N-glycan levels were assayed by FACS for (a) CD25^+^CD4^+^ cells cultured for 24 h with or without anti-CD3/28 beads in the presence of rhIL-2 (b) Naïve CBA splenocytes gated on CD25^−^CD4^+^ and CD25^+^CD4^+^ T cells (c) CD25^+^CD4^+^ cells incubated with either PBS (CD25^+^CD4^+^) or KIF (CD25^+^CD4^+KIF^) for 30 min followed by culture for 24 h with rhIL-2 + anti-CD3/CD28 beads (d) CD25^+^CD4^+^ cells incubated with either PBS (CD25^+^CD4^+^) or KIF (CD25^+^CD4^+KIF^) for 30 min followed by culture for 24 h with rhIL-2. The data presented are representative of 3 separate experiments.

Activation of mouse splenic CD4^+^ and CD8^+^ T cells leads to dramatic remodeling of N-glycans [9]. Indeed T cell activation results in increased N-glycosylation on cell surface proteins [3]. In order to assay whether the increased alpha-1,2-mannosidase mRNA associated with activation of Treg is accompanied with changes in the level of cell surface protein N-glycans, we stimulated CD25^+^CD4^+^ Treg *in vitro* and quantified N-glycosylation with Phaseolus vulgaris leucoagglutinin (PHA-L) which binds specifically to tri- or tetra-antennary complex type N-glycans with β1-6 linked branching [19]. Although the 177/DST tolerance induction protocol enriches for alloantigen-specific Treg, alloantigen reactive Treg cannot be distinguished from Treg with other specificities present in the pretreated mice [20]. CD25^+^CD4^+^ T cells purified from 177/DST pretreated mice were therefore stimulated polyclonally *in vitro* with CD3/CD28 beads to ensure uniform activation. Figure 2a shows that polyclonal activation of Treg is accompanied with an increase in N-glycan expression on the cell surface (resting –v- activated Treg: MFI 89 –v- 312). Interestingly, naïve Treg express more cell surface N-glycans than CD25^−^CD4^+^ cells (Fig 2b Treg –v- CD25^−^CD4^+^: MFI 99 –v- 29). These data were verified using FACS sorted CD4^+^GFP^+^ Treg from Foxp3 knockin mice [21] (data not shown).

In order to assess whether blocking alpha-1,2-mannosidase activity with KIF corresponds with decreased Treg cell surface N-glycosylation, cells were incubated with KIF for 30 min and after washing were activated for 24 hours with CD3/CD28 beads. Treg incubated with KIF (Treg^KIF^) had decreased surface N-glycan expression compared with cells treated with control PBS (Treg) (Fig 2c Treg^KIF^ –v-Treg: MFI 14 –v- 312). The decreased N-glycosylation was less pronounced in KIF-treated cells that were cultured for 24 h without anti-CD3/CD28 stimulation (Fig 2d Treg^KIF^ –v-Treg: MFI 66 –v-89) indicating that there is a faster turnover of N-glycosylated cell surface proteins in activated Treg.

### N-Glycosylation Is Not Required for Treg Suppression In Vitro

**Figure 3:**
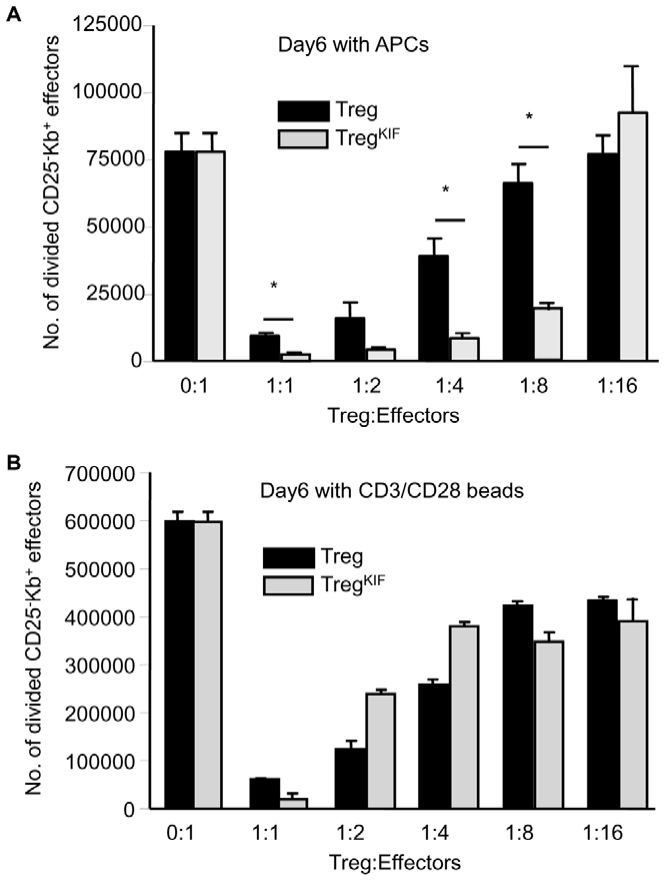
Treg^KIF^ retain their suppressive function in an *in vitro* MLR. Treg were purified from CBA mice and incubated for 30 mins with either PBS or KIF. Treg were cultured with CD3/CD28 beads with rhIL-2 for 24 hours and cultured at decreasing ratios with 1×10^5^ CFSE-labelled CD25^−^CD4^+^ effector K^b+^ T cells along with (a) 3×10^5^ irradiated B10.B10S.F1 splenocytes or (b) 1×10^5^ CD3/CD28 beads. After 6 days the cultures were harvested and numbers of divided CD25^−^CD4^+^ effector K^b+^ T cells were calculated by FACS. Error bars represent the standard deviation. Experiments were carried out in triplicate and data are representative of 3 separate experiments. * p<0.05.

It is well established that N-glycosylation of T cells alters their function [3], [22], [23], [24], [25]. Therefore we wanted to establish whether alpha-1,2-mannosidase function and hence N-linked glycosylation of proteins synthesized by Treg are required for their ability to suppress effector T cell responses. Due to the finding that cells pre-incubated with KIF had a more pronounced decrease in N-glycans on their cell surface after stimulation (fig 2c and 2d), Treg or Treg^KIF^ were cultured for 24 hours with IL-2 plus CD3/CD28 beads to allow the N-glycan profile of cells to change. Cells were then washed and co-cultured at decreasing ratios in an *in vitro* MLR with CFSE-labeled CBK CD25^−^CD4^+^ responder T cells and either CD3/CD28 beads or irradiated B10 APC stimulators. CBK cells are genetically identical to CBA, with the exception that they express the MHC I molecule K^b^ transgene which allows them to be distinguished from K^b−^ Treg. After 6 days in culture the number of responder T cells that had gone through at least one cell division was quantified. Figure 3 shows that the ability of Treg^KIF^ cells to regulate CD25^−^CD4^+^ cells was not impaired, when cells were stimulated with either irradiated B10 APC (Fig 3a) or CD3/CD28 expander beads (Fig 3b). Moreover, the ability of Treg^KIF^ to suppress to an alloantigen response was significantly better than Treg at ratios of 1∶1 (Treg 9672 +/− 1512–v- Treg^KIF^ 2566 +/− 1259 divided CD25^−^CD4^+^ cells; p<0.05) up to 1∶8 (Treg 66381 +/− 10220 –v- Treg^KIF^ 19210 +/− 3885 divided CD25^−^CD4^+^ cells; p<0.05). Hence, inhibiting N-glycosylation does not decrease the ability of Treg to regulate *in vitro,* and may even increase their suppressive capacity.

### Alpha-1,2-Mannosidase Inhibition Abrogates Treg Adherence In Vitro

N-glycosylation of many cell-surface molecules orchestrates their ability to bind to their respective ligands. Indeed inhibition of alpha-1,2-mannosidase with an alternative inhibitor deoxymannojirimycin results in decreased adherence of human lymphocytes to endothelial cells [26] suggesting that alpha-1,2-mannosidase and hence correct N-glycosylation regulates adherence.

**Figure 4:**
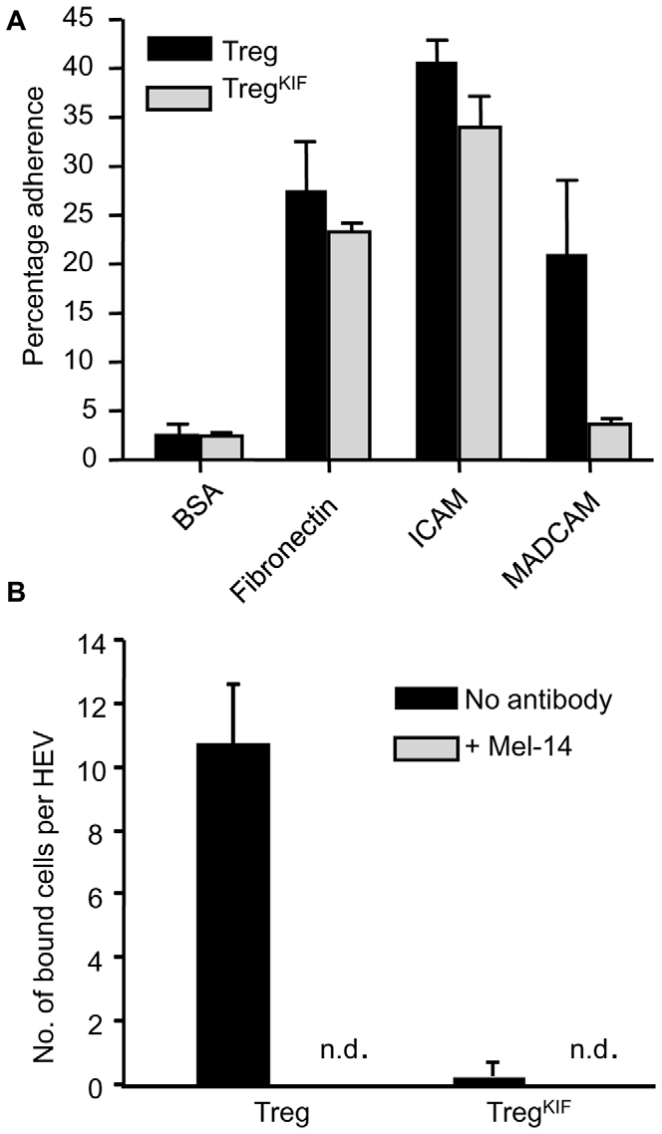
Adherence of Treg to various substrates after pre-incubation with KIF. Treg were purified from CBA mice and incubated for 30 mins with either PBS or KIF. Cells were cultured with CD3/CD28 beads with rhIL-2 for 24 hours. (a) 2×10^4^ cells were plated on Maxisorp plates coated with various substrates. After 45 min non-adherent cells were removed by washing and the percentage of adherent cells was quantified using CellTitre Glo (Promega). The percentage adherent cells after pre-incubation of Treg with PBS (Treg) or KIF (Treg^KIF^) is shown. (b) A modified Stamper-Woodruff protocol was performed. 10^5^ Treg were added to CBA ALN tissue sections and rotated over the sections for 45 min. Unbound cells were removed by washing in PBS. Treg bound to HEV were fixed. To block L-selectin binding, cells were pre-treated with Mel-14 antibody. The number of CFSE-labeled cells bound to HEV was counted blind. (n.d; not detected). Error bars represent the standard deviation. Experiments were carried out in triplicate and data are representative of 4 separate experiments. *p<0.05.

Our original identification of alpha-1,2-mannosidase levels associating with tolerance was identified in animals that had received a heart allograft [11]. In these animals Treg are likely to exit the peripheral blood to the peripheral lymphoid tissues and the graft where they can inhibit activation and effector function of immune cells with the potential to elicit graft destruction, in a process which requires Treg cell adherence to high endothelial venules (HEVs) or activated graft vessel endothelial cells. Others have identified the adherence molecules CD62L, VLA-4, and LFA-1 as mouse Treg markers whose expression is upregulated in axilliary LNs [27]. Therefore we assayed the ability of Treg or Treg^KIF^ to bind to ligands of CD62L, VLA-4, and LFA-1 in an *in vitro* adherence assay (Fig 4a). Treg were incubated on tissue culture plates coated with either BSA (negative control), fibronectin, ICAM-1 or MADCAM (binding by VLA-4, LFA-1 or CD62L respectively). Treg and Treg^KIF^ adhered to BSA at background levels and there was a trend towards less Treg^KIF^ binding to fibronectin although this was not statistically significant. A small but significant decrease was detected in the binding of Treg^KIF^ to ICAM-1 (Treg 41%±3.7, Treg^KIF^ 33%±5.5; p<0.05). The decreased ability of Treg^KIF^ to bind to MADCAM was more pronounced (Treg 15.7%±4, Treg^KIF^ 3.7%±0.6; p<0.05). These data show that Treg^KIF^ bind less efficiently to certain physiologically relevant ligands *in vitro.*

*In vivo* CD62L recognizes specific ligands on the HEVs of axillary lymph nodes and is considered the homing receptor for secondary lymphoid tissues [28]. CD62L is intensely glycosylated and its ability to bind to ligands *in vitro* depends on its glycosylation [29]. In order to confirm whether correct N-glycosylation of CD62L may facilitate Treg binding to HEV, we assessed the ability of Treg and Treg^KIF^ to bind axilliary LN sections in a modified Stamper Woodruff assay [30]. Approximately 10 Treg bound per HEV *in vitro* (Fig 4b). Virtually no Treg^KIF^ bound to HEV which was equivalent to incubating Treg with a CD62L-blocking antibody (Mel-14) (fig 4b). Interestingly, unlike Treg, high non-specific binding of Treg^KIF^ to the tissue sections at sites other than the HEV was observed, which was not inhibited by blocking CD62L (data not shown), suggesting that while inhibiting alpha-1,2-mannosidase impaired the ability of cells to bind to some ligands, the adherence of Treg^KIF^ to other ligands increases.

### Homing of Treg^KIF^ Is Disrupted *In Vivo* Which Allows Effector T Cell Priming in the dALN in Mice Receiving an Allogeneic Skin Transplant

We have previously shown that T- and B-cell deficient CBA Rag1^−/−^ mice reconstituted with BM3 T cells can reject donor skin allografts [31]. In these animals priming of BM3 T cells occurs in the draining axillary lymph nodes and can be detected by the proliferation of CFSE labeled BM3 cells at day 15 post-transplant [32]. Kinetic analysis of animals reconstituted with BM3 T cells together with Treg from tolerant mice, has shown that Treg are found in the draining lymph node at day 10 post-transplant where they prevent BM3 T cell priming [33]. Next, we used this established model to determine whether *in vitro* differences in Treg^KIF^ adhesion translate to *in vivo* differences in homing.

**Figure 5:**
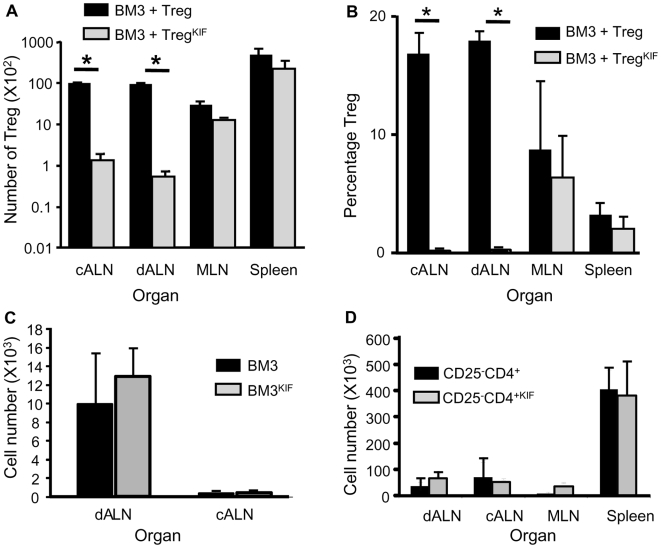
Ability of Kifunensine treated CD25+CD4+ cells to migrate *in vivo*. (a + b) CBA were pre-treated with an anti-CD4 mAb i.v. at days -28 and -27. At day -27, mice also received specific donor blood transfusion (DST). CD25^+^CD4^+^ Treg were purified from the spleen of these animals at day 0, and incubated for 30 min with either KIF or PBS. 5×10^5^ Treg were adoptively transferred into CBA-RAG^−/−^ animals along with 10^5^ CFSE labeled BM3 T cells. One day later mice received a B10 skin graft and lymphoid organs were harvested at day 10. Treg cell (a) numbers on a log scale and (b) percentage of total cells were analyzed by FACS. Data from n = 4 animals per group. Data is representative of 3 repeats. (5c) 10^5^ CFSE labeled BM3 T cells were adoptively transferred into CBA-RAG-/- animals. One day later mice received a B10 skin graft and axillary LNs were harvested at day 10. (5d) 10^5^ CD25^−^CD4^+^ T cells were adoptively transferred into CBA-RAG-/- animals. One day later mice received a B10 skin graft and peripheral lymphoid organs were harvested at day 10. The number of T cells was quantified by FACS. Data from n = 5 animals per group. Data are representative of 2 independent experiments. * p<0.01. Error bars represent the standard deviation.

**Figure 6:**
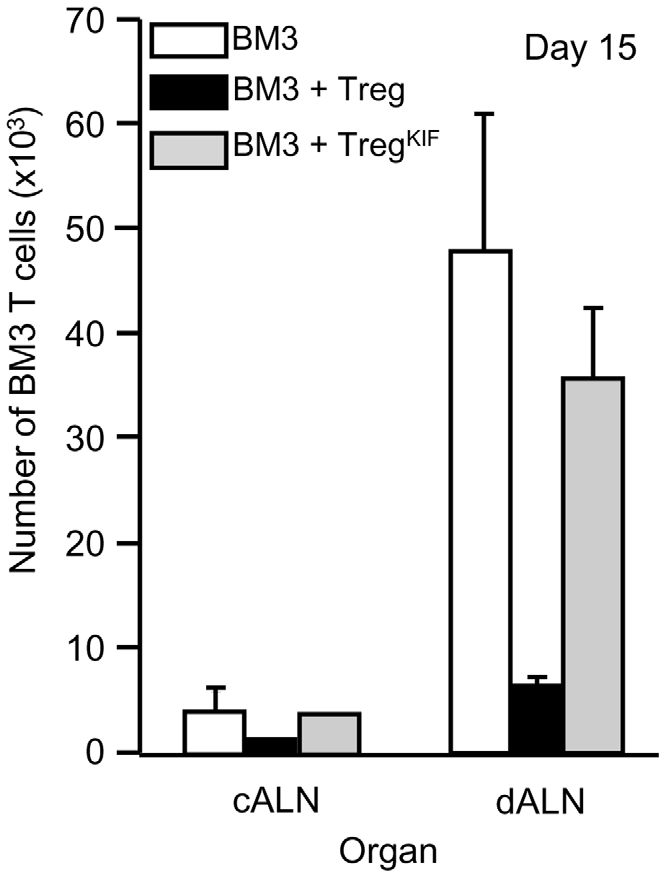
Ability of Treg^KIF^ cells to inhibit BM3 T cell priming is impaired. CBA were pre-treated with an anti-CD4 mAb i.v. at days -28 and -27. At day -27, mice also received specific donor blood transfusion (DST). Treg from these animals were purified at day 0, and incubated for 30 minutes with either PBS or KIF. 5×10^5^ Treg or Treg^KIF^ were adoptively transferred into CBA-RAG^−/−^ animals along with 10^5^ CFSE labelled BM3 T cells. One day later (day 0) mice received a B10 skin graft. Lymphoid organs were harvested at day 15. BM3 T cell numbers were analyzed by FACS (cells were gated on Ti98, TCRβ and CD8). n = 4 animals per group. Error bars represent the standard deviation. Data are representative of 3 experiments.

Treg or Treg^KIF^ generated following the 177/DST protocol were co-injected with 10^5^ CFSE labeled effector BM3 T cells into CBA-RAG^−/−^ mice. These mice received a B10 skin transplant one day later. 10 days post-transplant there were no significant differences in either the number or percentage of Treg and Treg^KIF^ in the mesenteric lymph nodes (MLN) and spleen (Fig 5a and 5b). However, there was a significant reduction in the number and percentage of Treg^KIF^ in both the draining axillary peripheral lymph nodes (dALN) (Treg^KIF^ 132±22, Treg 10271±193, p<0.01; Treg^KIF^ 1.97%±1.32, Treg 18.47%±3.06, p<0.05) and contralateral axillary peripheral lymph nodes (cALN) (Treg^KIF^ 155±21, Treg 9385±164, p<0.01; Treg^KIF^ 1.33%±0.6, Treg 17.14%±0.56, p<0.01). The ability of BM3 T cells and CD25^−^CD4^+^ cells to home to the axilliary peripheral LN was not inhibited at this timepoint (Fig 5c and 5d), although there were significantly less CD25^−^CD4^+^ cells in the ALN at day 5, following treatment with KIF (data not shown). Decreased Treg^KIF^ number in the dALN coincided with impaired ability of these cells to inhibit BM3 T cell priming (Figure 6). These data suggest that Treg^KIF^ are unable to migrate efficiently to the dALN, and as a result they cannot prevent BM3 T cell priming following an allogeneic skin graft.

### Treg^KIF^ Do Not Prevent Skin Graft Rejection Mediated by Effector T Cells In Vivo

**Figure 7:**
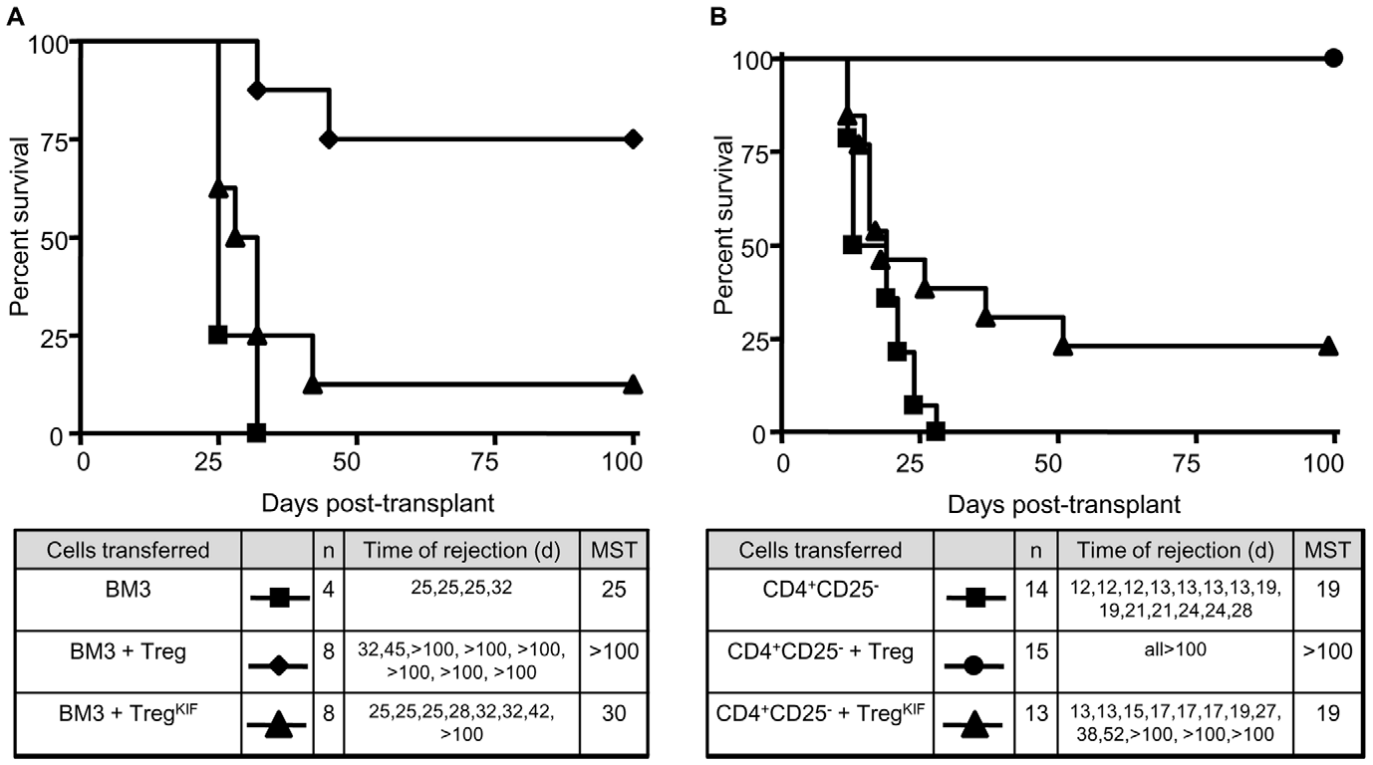
*In vivo* abrogation of regulation by inhibiting the function of alpha-1,2-mannosidase. The ability of Treg^KIF^ to prevent effector T cell mediated allograft rejection was assayed in an adoptive transfer model. (a) CBA RAG^-/-^ mice were reconstituted with either (a) 10^5^ BM3 T cells purified from BM3-RAG^-/-^ animals or together with either 3×10^5^ Treg or Treg^KIF^ from pre-treated mice, or (b) 10^5^ CD25^-^CD4^+^ syngeneic T cells from naïve animals or together with either 10^5^ Treg or Treg^KIF^ from pre-treated mice. The next day mice received an allogeneic skin graft from B10 mice. Skin graft survival was monitored over 100 days.

When CBA Rag1^−/−^ are reconstituted with BM3 T cells together with Treg from tolerant mice, prevention of BM3 T cell priming by Treg results in survival of a B10 skin graft in most animals [31]. In order to evaluate whether the inability of Treg^KIF^ to block BM3 priming results in their defective ability to protect allogeneic skin grafts from rejection, BM3 T cells were adoptively transferred into CBA-RAG^−/−^ recipients alone or along with either pre-treated Treg or Treg^KIF^. One day later these animals received a B10 skin graft and survival was monitored over 100 days. BM3 T cells mediated skin graft rejection with median survival time (MST) of 25 days. Adoptive transfer of Treg at a 3∶1 ratio prevented rejection in 6 out of 8 animals with skin grafts maintained for >100 days (Figure 7a). However animals adoptively transferred with BM3 T cells + Treg^KIF^ at the same ratio rejected with a MST of 30 days with only 1 out of 8 animals accepting their graft long-term.

To determine whether pre-treated Treg^KIF^ were also unable to prevent rejection mediated by a heterogeneous population of effector T cells, 1×10^5^ CD25^−^CD4^+^ T cells were adoptively transferred into CBA-RAG^−/−^ recipients alone or together with Treg or Treg^KIF^ at a ratio of 1∶1. CD25^−^CD4^+^ effector T cells alone mediated rapid skin graft rejection (MST 19d). In agreement with previously published data [13], co-transfer of CD25^+^CD4^+^ T cells from mice pre-treated with 177/DST results in long term survival of these grafts in CBA-RAG^-/-^ mice (MST >100d, n = 14), Figure 7b. In contrast, co-transfer of 1×10^5^ Treg^KIF^ resulted in long term survival in only 3 out of 15 mice, and the kinetics of rejection were similar to mice receiving effectors only (MST 19d). These data suggest that in most animals Treg^KIF^ cannot prevent B10 skin graft rejection by BM3 T cells or CD25^−^CD4^+^ cells. Incubation of BM3 T cells or CD25^−^CD4^+^ cells with KIF before adoptive transfer did not alter their kinetics of rejection (data not shown).

## Discussion

We have previously shown that the induction of immunological unresponsiveness to alloantigens *in vivo* correlates with an increased expression of alpha-1,2-mannosidase in both graft infiltrating leukocytes and peripheral blood mononuclear cells in various animal models of tolerance, whereas decreased levels of alpha-1,2-mannosidase serve as an indicator of rejection [11]. These tolerance induction protocols generate alloantigen reactive Treg [13], [16], [17] and we have shown here that when Treg encounter alloantigen a transient increase in alpha-1,2-mannosidase is observed. This suggests that alloantigen encounter results in changes in N-glycosylation of secreted and/or cell surface proteins by Treg, which was confirmed by N-glycan analysis of polyclonally stimulated Treg.

Interestingly polyclonal activation *in vitro* of mouse CD4^+^ splenocytes, which predominantly comprise CD25^−^ cells, results in decreased levels of alpha-1,2-mannosidase mRNA [11], suggesting that both the T cell subset and the nature of T cell activation may influence alpha-1,2-mannosidase expression.

Correct N-glycosylation could be blocked in Treg by inhibiting alpha-1,2-mannosidase activity with KIF, which gives rise to exclusively to high mannose structures [14], and we could detect a decrease in N-glycosylation of cell surface proteins, particularly in cells stimulated *in vitro* with CD3/CD28.

We and others have previously shown that inhibiting alpha-1,2-mannosidase results in altered T cell behavior [11], [24]. Despite decreased cell surface N-glycans, Treg^KIF^ maintained their ability to suppress effector cell proliferation in response to either polyclonal CD3/CD28 or alloantigen stimuli *in vitro*. Emerging data have identified several secreted Treg proteins that mediate their suppressor function. These include galectins which are proteins that can selectively kill effector T cells [34]. Galectins are not glycosylated [35] and therefore their production and function may not be affected by blocking alpha-1,2-mannosidase. We have established that IFN-γ production by Treg is important for their regulatory function *in vivo* [18]. Deficiency in Mgat5 which is an enzyme downstream of alpha-1,2-mannosidase in the N-glycan pathway, results in enhanced IFN-γ production in naive mouse CD4^+^ T cells when stimulated with plate-bound anti-CD3 and anti-CD28 antibodies [25] suggesting that inhibiting N-glycosylation might not inhibit cytokine production and could actually enhance it, which may explain the enhanced suppressor function of Treg^KIF^ *in vitro*. The N-glycosylation of IFN-γ has been well characterized, and in the absence of correct N-glycosylation IFN-γ may have an altered binding capacity [24] although the impact of this is unclear.

Glycosylation of cell surface proteins is integral to the ability of cells to interact with other cells and ligands. Treg^KIF^ maintained their ability to bind to fibronectin which is mediated by VLA-4 and VLA-5 [36] suggesting that alpha-1,2-mannosidase processing of these molecules is not essential for binding. The ability of Treg^KIF^ to bind to ICAM-1 was reduced suggesting that correct N-glycosylation of LFA-1 may facilitate Treg binding to ICAM-1. Interestingly, blocking MAN2C1, which is another enzyme in the N-glycosylation pathway downstream of alpha-1,2-mannosidase, results in increased binding of Jurkat human T cells to ICAM-1 [37]. Therefore the ability of Treg proteins to bind to ligands may depend on their terminal oligosaccharides. Together these data suggest that the effect of blocking N-glycosylation is both stage- and cell type-dependent and the process involves complexity which may be explained by differences in surface adherence molecule expression between cell types. The reduced ability of Treg^KIF^ to bind MADCAM *in vitro* was most pronounced. Lymphocytes interact with MADCAM via CD62L and α4β7 (CD103) [38], [39], which are both expressed on Treg [40]. Whilst very little is known about N-glycosylation of α4β7, CD62L has been shown to be intensely N-glycosylated [29]. Modification of CD62L N-glycosylation *in vitro* results in altered CD62L binding to ligands [29]. *In vivo* CD62L recognizes specific ligands on the HEVs of ALN and is considered the homing receptor for secondary lymphoid tissues [28]. Binding of CD62L to HEVs facilitates T lymphocyte rolling which precedes adherence and extravasation of cells into the ALN. The impaired binding to HEV by Treg^KIF^ was similar to Treg that were blocked with an anti-CD62L blocking antibody, suggesting that Treg^KIF^ may not be able to bind to HEV due to changes in N-glycosylation of CD62L. Interestingly Treg^KIF^ bound with high frequency to non-HEV ALN cells. Others have shown that inhibiting alpha-1,2-mannosidase function increases CD44 binding to hyaluronan (HA)[41]. Therefore differences in N-glycosylation may alter the range of ligands that Treg bind to, which facilitates trafficking of activated Treg.

Blocking CD62L *in vivo* dramatically decreases migration of mouse T cells to ALN [42]. Blocking alpha-1,2-mannosidase also impaired migration to ALN in CBA Rag1^–/–^ mice reconstituted with Treg^KIF^, suggesting that alpha-1,2-mannosidase and hence correct N-glycosylation of Treg is required for them to bind ALN HEV and exit the peripheral blood at these sites. Interestingly, the ability of Treg^KIF^ to home to the MLN and spleen was unaffected. CD62L on T cells binds to receptors such as MADCAM, GLYCAM and CD34 that express sialyl Lewis^a^ and sialyl Lewis^x^ epitopes. These epitopes are constitutively expressed on the HEV in ALN [43]. Data from CD62L-deficient mice has shown that while Treg numbers in the ALN are significantly lower, numbers in the MLN and spleen are similar to wild-type [44], which suggests that other adhesion proteins may mediate homing to these organs and may explain the differences we observe *in vivo*. Additionally, treatment of ALN with bacterial sialidases eliminates attachment of lymphocytes to the HEV whereas treatment of Peyer's patches do not [45]. This indicates that distinct mechanisms control endothelial cell attachment at different sites and may explain the distinct requirement of correct N-glycosylation of Treg for homing to the ALN but the not MLN. Chemokine receptors that are expressed on Treg are also essential for homing to lymphoid organs [46], [47]. Blocking chemokine receptors with Pertussis toxin injection results in reduced Treg numbers in the spleen [48]. Because we have observed normal homing of Treg^KIF^ to the spleen and MLN we have not investigated chemotaxis in our study, but we cannot rule out that this may contribute to the homing impairment of Treg^KIF^.

CD4^+^ and CD8^+^ T cells are similarly dependent on CD62L expression for migration into lymphoid tissues [49]. However, in contrast to Treg, following KIF treatment, at day 10 normal numbers of BM3 T cells and CD25^−^CD4^+^ T cells were detected in the ALN of reconstituted CBA Rag1^−/−^, suggesting that the requirement for N-glycosylation for homing may differ in these cells. This is supported by the finding that effector CD8^+^ T cells can home to reactive ALN in the absence of CD62L [50].

Emerging data suggests that homing of Treg is essential for their function [51] [52]. We have demonstrated here that alpha-1,2-mannosidase facilitates the trafficking of Treg to the ALN where they can prevent BM3 CD8^+^ T cell priming and hence skin graft rejection. Alpha-1,2-mannosidase may also be essential for homing of alloantigen reactive Treg to transplanted cardiac allografts in tolerant animals as the CD62L ligand, sialyl Lewis, is induced on the endothelium of heart allografts that are undergoing rejection [43]. This would explain the elevated alpha-1,2-mannosidase detected in graft infiltrating leukocytes from tolerant animals [11]. However we have not determined the contribution of Treg to the alpha-1,2-mannosidase signal detected in this population of leukocytes.

During lymphoid reconstitution, homeostatic proliferation occurs [53]. In our adoptive transfer system, Treg and Treg^KIF^ undergo homeostatic proliferation and activation after reconstitution of CBA Rag1^–/–^ animals. In this system, inhibition of alpha-1,2-mannosidase by KIF is transient, as by 15 days post-transplant normal numbers of Treg^KIF^ are present in the ALN and they have the correct N-glycan signature (data not shown). Interestingly CD25^−^CD4^+^ T cells pretreated with KIF have reduced numbers in the ALN at day 5 but this has reached control numbers by day 10. Given the finding that 1 out of eight, and 3 out of 15 animals reconstituted with Treg^KIF^ together with BM3 T cells or CD25^−^CD4^+^ cells respectively accept their grafts long-term, the fine balance between Treg and effector T cells in the dALN during the early immune response to the allogeneic skin graft may tip the balance from tolerance to rejection.

Treg are not the only immune cells to express alpha-1,2-mannosidase. Maturation of tolerogenic immature dendritic cells is accompanied by a more than 13 fold reduction in alpha-1,2-mannosidase mRNA, suggesting that the N-glycosylation of DCs coincides with their ability to stimulate an effector or tolerance response [54]. CD8^+^ T cells also express alpha-1,2-mannosidase mRNA to a similar level as CD4^+^ T cells [11]. Therefore alpha-1,2-mannosidase function may play an important role in many cell types for controlling the immune response to allografts and may provide a useful biomarker of tolerance. Indeed measurement of the alpha-1,2-mannosidase mRNA levels as a ratio with FOXP3 in peripheral blood leukocytes of human renal transplant recipients on various immunosuppressive regimes can distinguish drug-free tolerant patients from chronically rejecting patients (Hernandez-Fuentes et al, submitted).

Our findings may also have implications for the clinical immunosuppressive regimes currently employed. Mycophenolate Mofetil (MMF) is an anti-proliferative drug currently administered in some centers after transplantation. MMF treatment leads to a decrease in both the expression and glycosylation of some adhesion molecules by depleting guanosine nucleotides [55]. Rats that have received MMF have less upregulation of adhesion molecules in allogeneic kidney grafts which results in less lymphocyte binding [56]. Additionally, treatment of human CD4^+^ and CD8^+^ T cells with MMF results in decreased adhesion and transendothelial cell migration [57]. The specific effects on Treg are currently unclear.

In summary, we have shown here for the first time that the N-glycan profile of Treg changes upon activation by alloantigen and is integral to their function *in vivo*. Many groups are currently interested in expanding alloantigen-specific Treg *ex vivo* in order to return these cells to patients receiving an allograft [58]. In order to facilitate this, many studies have focused on identifying cell surface markers that correlate with the regulatory function of T cells. Our data suggests that it will also be important to consider the N-glycan profile of Treg as this may influence the ability of Treg to home correctly *in vivo* to sites where they mediate suppression, regardless of their ability to suppress in an *in vitro* MLR.

## Materials and Methods

### Mice

CBA.Ca RAG-1 knockout (CBA RAG^−/−^; H2^k^) mice were a gift from Dr. D. Kioussis (Mill Hill, London, U.K.). BM3 TCR-transgenic mice (BM3; H2^k^) [59] and CBK mice (H2^k^+K^b^ as a transgene) were a gift from Prof. A.L. Mellor (Institute of Molecular Medicine and Genetics, Augusta, GA). BM3 mice were crossed to a CBA RAG^−/−^ background for these studies, therefore all of their CD8^+^ T cells are specific for the MHC I K^b^ molecule. CBA.Ca (CBA; H2^k^) and C57BL/10 (B10; H2^b^) mice were originally purchased from Harlan Olac. All mice were bred in the SPF facility, Biomedical Services, JR Hospital, Oxford. All experimental mice were sex- and age-matched aged between 6 and 12 wk at the time of the first procedure. All mice were bred and used in accordance with the Animals (Scientific Procedure) Act 1986 of the UK.

### Reagents and Monoclonal Antibodies

Non-depleting anti-CD4 (YTS 177.9) and anti-CD8 (YTS 169) [60] hybridomas were kindly provided by Prof H. Waldmann (Sir William Dunn School of Pathology, Oxford, UK). TIB120 (anti-MHC II), M1/70 (anti- Mac-1), and R3-6B2 (anti- B220) hybridomas were obtained from ATCC, Manassas, VA. All antibodies were produced *in vitro*, purified by chromatography and confirmed to be endotoxin free before use *in vivo*.

### Pre-Treatment Protocol

CBA mice received anti-CD4 mAb YTS177.9 (177) plus B10 (donor-specific transfusion (DST)) blood intravenously as previously described [20]. Spleens, ALN and MLN were harvested on d 0. Cells from these 177/DST pre-treated animals are termed “pre-treated”.

### Purification of T Cells by FACS Sorting, for Real-Time PCR Analysis of Gene Expression

Spleens were harvested from mice and CD4^+^ cells were purified as previously described [61]. Cells were stained for cell surface CD4 and CD25 and were selected using a FACS aria flow cytometer (BD Biosciences). Cell purity was analyzed by flow cytometry and cells were typically >95% pure. The level of alpha-1,2-mannosidase was quantified by real-time PCR as described previously [11].

### Purification of Treg and Responder CD4+ Cells

CD25^−^CD4^+^ responder T cells were isolated from LN and spleens of naive CBA, and CD25^+^CD4^+^ Treg were obtained from LN and spleens of either naïve or pre-treated mice. CD4^+^ cells were purified, as described previously [61]. CD25^+^ and CD25^−^ cells were purified using a CD25 microbead kit, following manufacturer's instructions (Miltenyi Biotec Ltd., Bisley, U.K.). Purity was determined by flow cytometry. Cells were typically >90% pure.

### Measurement of Surface N-Glycosylation Levels

Cells were resuspended in PBS (Oxoid, UK) containing 1 µg/ml biotinylated PHA-L (Vector Labs, Burlingame, CA). Cells were then stained with anti-CD25-FITC, anti-CD4-APC (Insight Biotechnology) and streptavidin-PE (BD Biosciences), acquired by flow cytometry.

### Inhibition of Alpha-1,2-Mannosidase

For pre-incubation with KIF (Toronto Research Chemicals, Canada), cells were resuspended in PBS+2% FCS (P.A.A. Laboratories GmBH) containing 40 µM KIF (Treg^KIF^) or control PBS only (Treg). Cells were incubated at room temperature for 30 min.

### *In Vitro* Suppression Assay

CD25^−^CD4^+^ “responder” cells were purified from CBK mice. Responder cells were labeled with 5 µM CFSE (Invitrogen) as previously described [32]. Cells were cultured in U-shaped 96-well plates (Corning Costar) at with various ratios of Treg from naïve CBA. Culture medium was composed of RPMI 1640 (Invitrogen) supplemented with 10% FCS (both P.A.A. Laboratories GmBH), 2 mM L-glutamine (Invitrogen), 0.5 mM 2-ME (Sigma-Aldrich), and 100 U/ml each penicillin and streptomycin (Invitrogen). Responder cells were stimulated with either CD3/CD28 T Cell Expander beads (Invitrogen), or irradiated B10 splenocytes. Potential binding of Fc receptors were blocked with Fc block (BD Biosciences), and cells were stained with anti-CD4-APC (Insight Biotechnology), anti-K^b^-biotin (BD Biosciences) followed by streptavidin-PE (BD Biosciences). Viability was determined using 7-AAD (BD Biosciences). Samples were acquired by flow cytometry. The total number of cells after 6 d was calculated by adding a fixed number of 6 mM synthetic fluorescent beads (CaliBRITE; BD Biosciences) to each sample.

### *In Vitro* Adhesion Assay

Treg were incubated for 24 h with CD3/CD28 T Cell Expander beads. 2×10^4^ cells / well were transferred to Maxisorp plates pre-coated with either 2.5% BSA (Sigma), bovine fibronectin (Sigma), 20 µg/ml rICAM1-Fc (R&D) or 20 µg/ml rMADCAM-1/Fc (R&D). Cells were centrifuged at 100 g for 1 min. After 45 min wells were gently washed 5× with PBS and resuspended in PBS. CellTitre Glo (Promega, UK) was added to the wells and samples were transferred to an opaque white 96-well plate (BD Biosciences). Values were measured using a luminometer (Lucy; Anthos Eugendorf Austria). Standard numbers of CD25^+^CD4^+^ cells were measured in parallel to generate standard curves in order to determine the percentage adherence.

### Stamper-Woodruff Assay

A modified Stamper-Woodruff protocol [30] was performed. ALN were collected from 5 CBA, pelleted together and snap-frozen in liquid nitrogen. Frozen tissue samples were cut to a thickness of 7 µm and allowed to air-dry on microscope slides for 2 h. A hydrophobic circle was drawn around each section, and the sections were placed on an orbital shaker at 4°C at 80 rpm.

Treg were cultured for 24 h with CD3/CD28 beads + hrIL-2 and were labeled with 5 µM CFSE. 100 µl of Treg (10^5^) were added and rotated over the sections for 45 min. Unbound cells were removed by washing in PBS. Treg bound to HEV were fixed by placing sections in cold 1.5% glutaraldehyde overnight. To block L-selectin binding, cells were pre-treated with Mel-14 antibody at 20 µg/ml for 30 min. Pretreated cells were rotated over the sections as described above. The number of CFSE-labeled cells bound to HEV was counted blind.

### *In Vivo* Tracking Assay

BM3 CD8 cells were purified and labeled with CFSE as previously described [33]. 10^5^ BM3 cells were adoptively transferred into CBA Rag1^−/−^ mice along with 5×10^5^ Treg. Unless stated, mice received a B10 skin graft 1 day later. At days 5, 10 or 15 post-transplant, a single-cell suspension was prepared from spleen, MLN, and draining or contralateral ALN. For analysis of BM3 tracking, cells were processed as previously described [32]. For analysis of CD4 cells tracking, cells were stained with anti-CD4-APC, anti-TCRβ-PE and anti-CD25-bio. All samples were then stained with Streptavidin-PECy5 (BD Bioscience) and acquired by flow cytometry. A fixed number of 6 mM synthetic fluorescent beads (CaliBRITE beads; BD Biosciences) were added to each sample in order to determine cell numbers.

### Adoptive Transfer and Skin Graft Survival Assay

T cell–deficient CBA Rag1^–/–^ mice were reconstituted i.v. with syngeneic fractionated T cells. One day after reconstitution, mice received a B10 skin graft as previously described [12]. Grafts were monitored and rejection was defined by complete destruction of the skin.
